# Investigation of Bioaccumulation and Human Health Risk Assessment of Heavy Metals in Crayfish (*Procambarus clarkii*) Farming with a Rice-Crayfish-Based Coculture Breeding Modes

**DOI:** 10.3390/foods11030261

**Published:** 2022-01-19

**Authors:** Fangjun Peng, Jiawen Li, Zhiyong Gong, Bing Yue, Xueli Wang, Anne Manyande, Hongying Du

**Affiliations:** 1 College of Food Science and Technology, Huazhong Agricultural University, Wuhan 430070, China; pfangjun@163.com; 2 National R & D Branch Center for Conventional Freshwater Fish Processing, Wuhan 430070, China; 3 QianJiang Public Inspection and Testing Center, Qianjiang 433100, China; neverlove1983@163.com; 4 Key Laboratory for Deep Processing of Major Grain and Oil of Ministry of Education, Wuhan Polytechnic University, Wuhan 430023, China; gongzycn@whpu.edu.cn; 5 Department of Scientific Education & International Cooperation, China National Center for Food Safety Risk Assessment, Beijing 100022, China; yuebing@cfsa.net.cn; 6 School of Mathematics and Statistics, Beijing Technology and Business University, Beijing 100048, China; xlwang@btbu.edu.cn; 7 School of Human and Social Sciences, University of West London, Middlesex TW8 9GA, UK; Anne.Manyande2@uwl.ac.uk

**Keywords:** crayfish (*Procambarus clarkii*), heavy metal, ICP-OES, risk assessment, bioaccumulation

## Abstract

Due to the rapid development of the crayfish (*Procambarus clarkii*) industry in Chinese catering, people are paying more attention to the bioaccumulation of heavy metals in crayfish. To evaluate the health risks associated with the consumption of crayfish, nine types of heavy metals in both crayfish and abdominal muscles of crayfish were investigated. Crayfish samples were collected from rice-crayfish-based coculture breeding modes from different areas located in the middle and lower reaches of the Yangtze River. The average concentrations of heavy metals in the whole crayfish were much higher than the abdominal muscle of crayfish. The estimated daily intake (EDI) of heavy metals in the abdomen of crayfish was calculated to assess the noncarcinogenic risk and the overall noncarcinogenic risk including the target hazard quotient (THQ), the hazard index (HI) and carcinogenic risk (CR). The results of the present study showed that the consumption of crayfish may not present an obvious health risk to human associated with heavy metals. However, the THQ values of As in the abdominal muscles of crayfish for adults in EnShi (ES) and children in JiaYu (JY) should be of concern due to the higher contribution to the potential health risks of crayfish compared to other metals. Through X-ray photoelectron spectroscopy (XPS) detection of heavy metal As, it is found that As in crayfish culture environment mainly exists in the form of As^3+^.Therefore, the quality and quantity of crayfish consumption should be moderated to prevent the bioaccumulation of As. The results indicate that crayfish cultured in different areas may have similar pollution levels and/or emissions from the same pollution sources.

## 1. Introduction

Large amounts of heavy metals are disposed into rivers due to rapid development in industry and agriculture. These heavy metals can be transferred to the water, sediment, and aquatic food chain and amplified, leading to the accumulation of heavy metals in aquatic products, such as red swamp crayfish (*Procambarus clarkii*) [1,2,3,4]. Heavy metal pollution is a serious environmental issue in many countries and caused by agricultural and industrial waste discharged into the river or sea. Since heavy metals are easily bioabsorbed and bioaccumulate, they pose a potential risk to human health through eating the contaminated food [5]. Heavy metals have carcinogenic effects, which may lead to mental health problems or reduced cognitive development in children and increased cardiovascular diseases in adults; in addition, they may cause kidney and reproductive dysfunction [6]. Therefore, the detection of heavy metal contents in crayfish is very important for food safety and risk assessment.

With the increasing incidence rate of human cancers worldwide, greater efforts should be made to prevent and control cancer [7]. In recent years, the scientific community has become increasingly interested in understanding the potential health risks of human exposure to single or groups of pollutants from natural or man-made sources [5,8]. Aquatic products that are rich in protein, fatty acids, carbohydrates, vitamins, and important microelements are being consumed more by human beings [9]. As a popular aquatic product, crayfish are nutritious and delicious and rich in protein and vitamins, which play an important role in supporting human health [10,11]. Furthermore, the price of crayfish is cheaper than that of other shrimp since it can be cultured in rice-crayfish-based coculture breeding modes, which reduces the cost of breeding. Therefore, crayfish have been increasingly favored by consumers [12]. In recent years, Hubei has become the largest crayfish-producing province in China, especially in the middle reaches of the Yangtze River [13]. Hubei spicy crayfish, a typical snack food in Hubei Province, is renowned both in China and abroad on account of its great taste. However, crayfish also face the risk of heavy metal contamination, as aquatic products tend to accumulate heavy metals from the aquaculture environment and spread it upward along the food chain [14,15].

Previous studies on the quality and safety of crayfish in China showed that the accumulation of heavy metals in crayfish could pose a threat to human health [16,17]. For instance, Wang et al. conducted a comprehensive study of the five heavy metals in four aquatic products in China during 2015–2017 [9] and reported that crayfish had the highest concentration of heavy metals harmful to human health. The exposure risk for Eurasian otter habitat was also measured, and it was concluded that exposure to metals through crayfish consumption may prevent or slow down the reproduction of otters [18]. The bioaccumulation of heavy metals in crayfish was studied under different environmental pollution conditions, and low or high levels of heavy metals in the abdominal muscles of crayfish were found to be related to pollution sites [19]. The first large-scale assessment of the health risk associated with heavy metals in crayfish demonstrated that there might be a health risk for frequent consumers, highlighting the importance of considering ingestion rates [20]. Furthermore, the distribution of heavy metal contents in different tissues of wild and cultured crayfish was investigated, including the exoskeleton, abdominal muscle, gills, and hepatopancreas [21]. However, the health risk assessment of heavy metals determined in crayfish cultured in different geographical locations, such as the largest crayfish farming in Hubei Province, remains unclear.

In this study, crayfish samples cultured in rice-crayfish-based coculture breeding modes were collected from 10 different cities in the middle and lower reaches of the Yangtze River Basin of Hubei Province. The spatial distribution of heavy metals in crayfish and the human health risks associated with the consumption of crayfish abdominal muscles were investigated to screen or evaluate the external environment and establish an effective risk prediction.

## 2. Materials and Methods

### 2.1. Sample Collection

**Figure 1:**
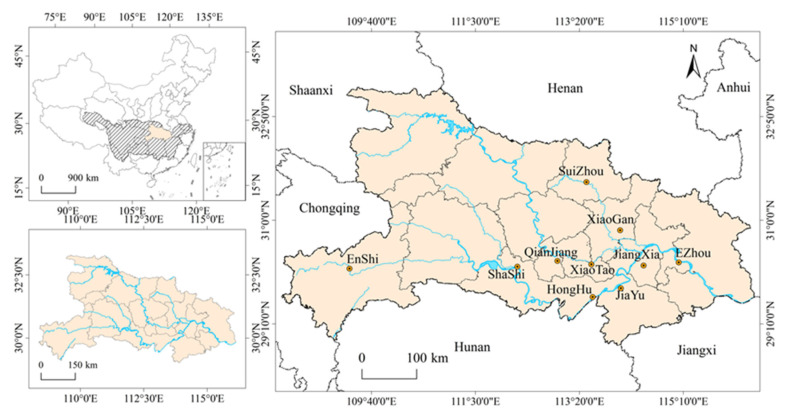
Maps of the province of Hubei indicating the study areas.

In the current research, the study sites consist of 10 cities along the Yangtze River Basin in Hubei Province, namely, XianTao (XT), Ezhou (EZ), SuiZhou (SZ), HongHu (HH), JiangXia (JX), JiaYu (JY), QianJiang (QJ), XiaoGan (XG), ShaShi (SS), and EnShi (ES). The geographical regional distribution map of the sample collection areas is shown in Figure 1, and the longitude and latitude information of these regions are illustrated in Table S1. The red swamp crayfish samples were collected from rice-crayfish-based coculture breeding modes in areas a slight distance from the road in May 2021, and each selected farmland had nonpoint source pollutants, reflecting the impact of agricultural nonpoint source pollution. Twelve crayfish of similar size were obtained from each farmland. Each crayfish was weighted after cleaning. All samples were stored in a clean self-sealing bag, marked, and then refrigerated for future determination of heavy metal concentrations.

### 2.2. Chemicals and Reagents

Here, all chemicals, heavy metal stock solutions, multielement solutions and acids were of analytical grade and obtained from Merck Co. (Darmstadt, Germany).

### 2.3. Sample Digestion and Analysis

The samples needed to be digested and dissolved before taking measurements [9]. Briefly, 12 crayfish were randomly divided into two equal groups. In one group, six crayfish were dissected to obtain abdominal muscles, while in the other group, they were not dissected. All samples were dehydrated completely at 80 °C for several hours until a constant weight was achieved and then stored in self-sealing bags. The dried samples were ground to form powder, and 0.5 g of homogenized crayfish powder was added into a digestion container filled with 65% HNO_3_ (6 mL) and 30% H_2_O_2_ (1 mL). After 30 min, the digestion container was transferred into an autoclave and heated at 120 °C for 10 h, allowed to cool to room temperature, transferred to a volumetric flask and diluted with 1% HNO_3_ to 25.0 mL for testing.

To investigate the concentrations of the nine heavy metals, 1.0 mL of the digested solution was detected by an inductively coupled plasma atomic emission spectrometer (ICP-OES PerkinElmer optima 8000, Waltham, MA, USA) equipped with an ultrasonic nebulizer (CETAC Technologies, U-5000AT, Omaha, NE, USA) following a former detecting protocol [22]. Commercially produced standard solutions (1000 mg L^−1^, ICP multielement standard solution IV CertiPUR^®^, VWR Merck Chemicals and Reagents, Bengaluru, Indian) in nitric acid were utilized to prepare the appropriate elemental calibration standards. For analysis of the spectra of As, Cd, Ba, Cr, Hg, Cu, Ni, Mn, and Pb, we try to select the spectral line with no or less interference, the highest sensitivity and appropriate signal strength. At the beginning of the test, each element shall be measured by five different spectral lines, and then the best element analysis spectral line shall be determined according to the actual situation.

### 2.4. Quality Assurance and Quality Control

Concentrations of elements were determined in triplicate; the repeatability of ICP-OES measurements was generally ≥ 97%. Quality assurance (QA) and quality control (QC) of elements analysis were performed using certified reference material (CRM): GSB 04-1767-2004 (National standard sample) and duplicate measurements. The CRM was detected with every ten samples to ensure the accuracy of the procedure. The heavy metal standards were spiked and digested to check the recovery, and the blank sample were not spiked. The detection limits for As, Cd, Ba, Cr, Hg, Cu, Ni, Mn, and Pb, are 0.0001 mg/L(ppm). There were three samples in every detection.

### 2.5. X-ray Photoelectron Spectroscopy (XPS)

The XPS analysis was utilized to detect the chemical states of as in the crayfish. XPS was conducted using an ESCALAB 250Xi photoelectron spectrometer (Thermo Fisher Scientific, Carlsbad, CA, USA). Al K Alpha (1486.6 eV) was the X-ray source set at 200 W, and a pass energy (50 eV) was set for the high-resolution scan.

### 2.6. Health Risk Assessment

The human body could accumulate the heavy metals from crayfish after eating, which is considered to be a unique and important mode of exposure [23]. The target hazard quotient (THQ) was utilized to evaluate the noncarcinogenic health risk by the combination of the oral reference dose (RfD) and daily intake (EDI), while the carcinogenic health risk was assessed by the carcinogenic risk (CR).

#### 2.6.1. Detection of Estimated Daily Intake (EDI) of Metal

Depending on the concentration of heavy metals in aquatic products and the daily consumption of aquatic products, the EDI of nine heavy metals was obtained from the following equation [24]:

(1) $$\mathrm{EDI}=\frac{C\times \mathrm{FIR}}{\mathrm{BW}}$$

where, according to [25]: C is the heavy metal concentration in the tissue (mg kg^−1^, dry weight, a conversion factor-4.8 was utilized to convert the wet weight to dry weight [21]); FIR is the ingestion rate (assuming a daily meal size of abdominal muscle: children—72 g; adults—168 g) [26], and BW is the average body weight (adult: 70 kg [21], seven-year-old children [10]: 16 kg). In addition, EDI were compared with the corresponding provisional daily intakes (PTDI) recommended by the FAO/WHO Joint Expert Committee on Food Additives and the Committee on Food and Nutrition (2004) [27].

#### 2.6.2. Detection of Target Hazard Quotient (THQ)

The THQ was obtained from heavy metal concentrations and daily consumption of the aquatic products. It should be considered that the heavy metal gave a potential risk to human health if THQ value was bigger than one; otherwise, the risk could be ignored [10]. Here, the THQ was calculated with the following formula [9]:

(2) $$\mathrm{THQ}=\frac{\mathrm{EF}\times \mathrm{ED}\times \mathrm{EDI}}{\mathrm{RfD}\times \mathrm{AT}}\times 10^{-3}$$

where EF is the exposure frequency (365 days/year), ED is the exposure duration (70 years), EDI is the estimated daily intake of the heavy metal from the abdominal muscle of crayfish (mg kg^−1^, dry weight), RfD is the safe reference oral dosage of heavy metals (µg kg^−1^ day^−1^; As, 0.3; Ba, 200; Cd, 1; Cr, 1500; Cu, 40; Mn, 140; Ni,20; Pb, 4) [5,28] and AT represents the average exposure time (365 days/year times by number of exposure years). 

To evaluate the overall noncarcinogenic risk from multiple substances and exposure routes, the hazard index (HI) was calculated from the following equation [29]:

(3) $$\mathrm{HI}={\mathrm{THQ}}_{\left(\mathrm{As}\right)}+{\mathrm{THQ}}_{\left(\mathrm{Ba}\right)}+{\mathrm{THQ}}_{\left(\mathrm{Cd}\right)}+{\mathrm{THQ}}_{\left(\mathrm{Cr}\right)}+{\mathrm{THQ}}_{\left(\mathrm{Cu}\right)}+{\mathrm{THQ}}_{\left(\mathrm{Hg}\right)}+{\mathrm{THQ}}_{\left(\mathrm{Mn}\right)}+{\mathrm{THQ}}_{\left(\mathrm{Ni}\right)}+{\mathrm{THQ}}_{\left(\mathrm{Pb}\right)}$$

HI values < 1 indicate that exposed individuals are less likely to suffer from adverse health effects, while HI values > 1 reflect that adverse health effects may occur [30].

#### 2.6.3. Evaluating the Cancer Risk (CR)

The lifetime CR of exposure to heavy metals was defined as the incremental probability of an individual developing cancer based on the cancer slope factor (CSF). The carcinogenic risk could be estimated from the value of CR, such as CR > 1 × 10^−4^: carcinogenic risk; 1 × 10^−4^ > CR > 1 × 10^−6^: acceptable carcinogenic risk, and CR < 1 × 10^−6^: negligible carcinogenic risk [31].

The CR was obtained from the following formula [28]:

(4) $$\mathrm{CR}=\frac{\mathrm{EF}\times \mathrm{ED}\times \mathrm{EDI}}{\mathrm{AT}}\times \mathrm{CSF}\times 10^{-3}$$

where CSF is the carcinogenic slope factor, and the CSF values are 1.5, 6.3, 0.5, 8.5 × 10^−6^ and 0.0085 mg kg^−1^ day^−1^ for As, Cd, Cr, Hg and Pb, respectively [9,21]. 

### 2.7. Principal Component Analysis (PCA)

The kernel idea of principal component analysis (PCA) is dimensionality reduction, which was conducted using the varimax rotated factor matrix method based on the orthogonal rotation criterion. PCA uses chemical element principal factor loading to identify source types. The PCA method was implemented with a home-made m-file code under the MATLAB/Simulink R2018a (The MathWorks, Inc., Natick, MA, USA).

### 2.8. Statistical Analysis

The statistical analysis was implemented in MATLAB R2018a and the mathematical data were analyzed using Excel 2019. Variability among the metals was set up as most significant at the 99% confidence level (*p* ≤ 0.01). ArcMap 10.2 software (ESRI-Environmental Systems Research Institute, Inc., Redlands, CA, USA) was utilized to draw the graphs.

## 3. Results and Discussion

### 3.1. Mean Concentrations of Heavy Metals in Crayfish and Abdominal Muscles of Crayfish 

Due to the health problems, the regulatory limits of the heavy metals in the different kinds of food were stipulated in China [32]. To estimate the quality control of the samples, the percentage recovery (%) of the heavy metals was determined by comparing the concentrated values of each metal from spiked and unspiked samples. The recoveries of metals in the samples were between 90–105%, which was in the range of acceptable mean percentage recoveries (70–110%) [10]. Thus, this verified the reliability of the current study. 

The heavy metal concentrations measured in crayfish from 10 different cities in Hubei Province are collected in Table S2. Among the samples, the highest concentrations of As (4.05 mg/kg), Mn (559.19 mg/kg), and Pb (0.65 mg/kg) were found in JX. The crayfish in JY had the highest concentrations of Cr (2.88 mg/kg), Cu (85.62 mg/kg), and Ni (1.60 mg/kg). The highest concentrations of Ba (244.70 mg/kg) and Cd (0.31 mg/kg) originated in HH, while the highest concentrations of Hg (0.05 mg/kg) were detected in QJ. The range and mean levels of the heavy metals measured in the abdominal muscles of crayfish are illustrated in Table S3. The table shows that the highest levels of As (1.27 mg/kg) and Ba (2.13 mg/kg) were found in JY and JX, respectively, whereas the lowest levels of As (0 mg/kg) and Ba (0.83 mg/kg) were collected from HH and ES, respectively. The highest concentrations of Cd, Cr, Cu, Mn, and Ni originated from samples in SS; however, the lowest levels of those heavy metals emerged from SZ, HH, HH, QJ, and HH.

Concentrations of the heavy metals in the whole body of crayfish are significantly higher than that in the abdominal muscles of crayfish. For example, As and Ni are approximately 800- and 46-fold higher in HH; Ba in JX is 180-fold higher; Cd and Pb in SZ are 66- and 64–202 fold higher, and Mn in EZ is 266-fold higher. According to the heavy metal residue analysis in crayfish and the abdominal muscle of crayfish, the heavy metal residues occurring in the environment are mainly concentrated in the head and exoskeleton of crayfish, which is consistent with previous studies [21,33]. The relatively low concentrations of Pb and Cd could be explained by the fact that the crayfish are mostly cultivated in rice-crayfish coculture breeding modes and exposed to heavy metals for a relatively short time [34]. Additionally, the concentrations of heavy metals in crayfish are generally within the range of heavy metals in aquatic products reported in China [35]. These results indicate that although there is a small amount of accumulated heavy metals, it is safe for local residents to eat abdominal muscles of crayfish from the studied areas [21].

### 3.2. XPS Measurement

**Figure 2:**
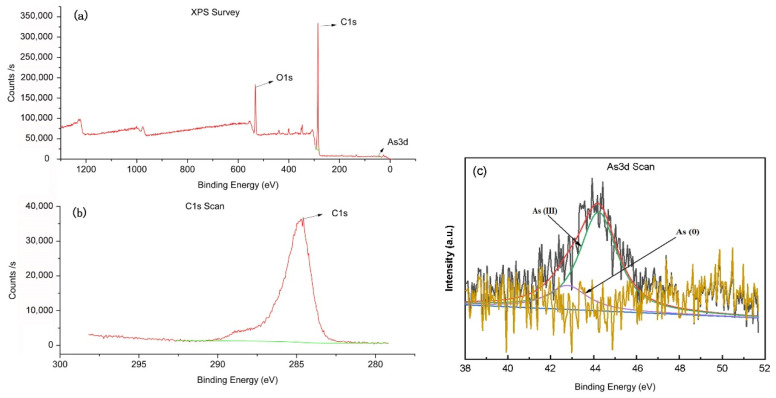
X-ray photoelectron spectroscopy (XPS) of crayfish showed different peak positions (**a**), (**b**) C_1s_ XPS survey spectrum, and (**c**) As_3d_ XPS survey spectrum.

In order to further investigate the occurrence form of heavy metal arsenic in crayfish, X-ray photoelectron spectroscopy (XPS) surface analysis technology [36] is applied, as shown in Figure 2. In the figure, there are two peaks of C_1s_ and As_3d_, 279.23–292.63 eV and 37.68–51.68 eV respectively. It can be seen from the XPS standard spectrum that the C_1s_ represented by the peak positions with electron binding energies of 284.80 and 43.92 EV are corrected as a reference to eliminate the influence of charging effect and the heavy metal As appears in the valence state of As^3+^. This result directly shows that As compounds containing As^3+^ and As in crayfish culture environment are enriched in crayfish [37,38,39,40]. The inorganic form of As is considered toxic and they exist mainly in trivalent (As^3+^, arsenite, 88.2%) state and nulvalent (As, 11.8%) state and the trivalent form are supposed to more toxic as compared to the pentavalent form [41].

### 3.3. Correlation Matrix Analysis (CMA)

**Figure 3:**
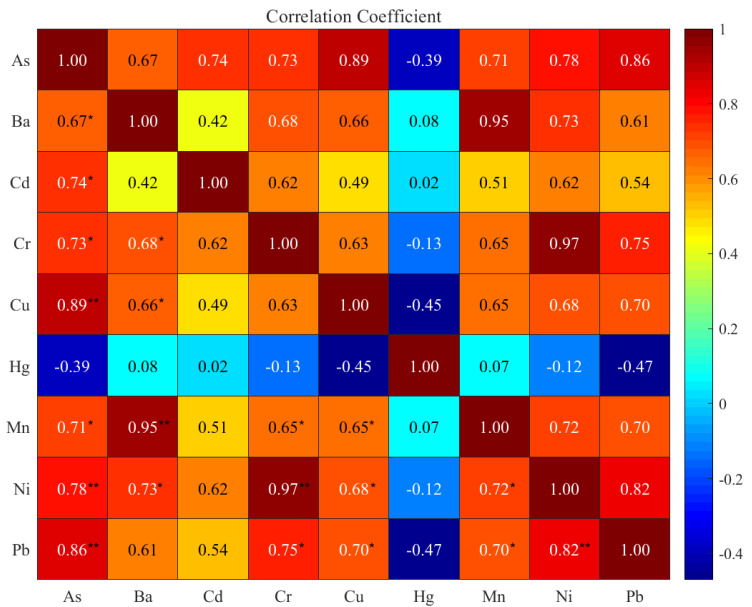
Pearson correlation analysis of heavy metals in the whole body of crayfish. The correlation coefficient is illustrated by the intensity of the colors, as shown by the color scale (Notes: *: *p* < 0.05, **: *p* < 0.01).

Results from Pearson correlation analysis of different metal concentrations in the whole body of crayfish are collected in Figure 3. Among the correlations, As-Cu (r: 0.89), As-Pb (r: 0.86), As-Ni (r: 0.78), Ba-Mn (r: 0.95), Cr-Ni (r: 0.97), and Ni-Pb (r: 0.82) reflect strong significant relations (*p* < 0.01). In addition, correlations revealed that the accumulation of the metals was closely related to the other metals in the whole crayfish (whole crayfish). This strong and significant positive correlation indicates that heavy metals in the whole crayfish (whole crayfish) from different geographical environments shared similar pollution sources and/or common background levels. In addition, Cd, Cu, and Pb were identified by another group of metals based on their significant positive correlations, but Hg did not show any significant correlations with these components. As was also significantly correlated with Ba, Cd, Mn, and Cr (*p* < 0.05), indicating that the accumulation of As in organisms is closely related to these associated metals [14]. Previous studies have reported that As, which is a nonessential element for the whole crayfish, tends to accumulate in crayfish tissues with increasing concentrations in the environment and exposure time [33,42]. Hg has also been shown to be a human carcinogen that has the potential to destroy ecological communities [43]. A negative correlation may indicate that the metals are from different sources or have nonchemical similarity.

### 3.4. Principal Component Analysis (PCA)

**Figure 4:**
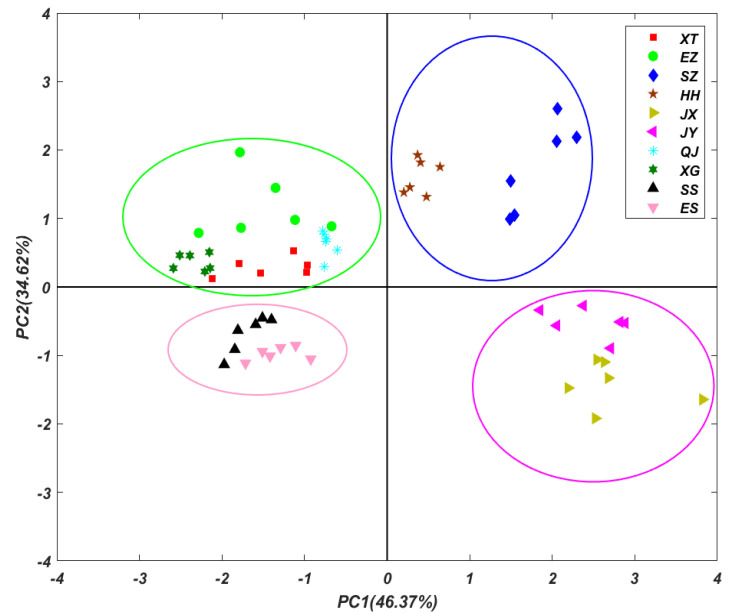
The scores of PC1 and PC2 in principal component analysis of crayfish (whole crayfish) in different areas along the Yangtze River Basin of Hubei Province.

To trace the source of heavy metals, PCA method was utilized to investigate the concentrations of different heavy metals in crayfish from different regions of Hubei Province [14]. The first two principal components (PCs) were obtained and illustrated to discriminate the samples from different areas (Figure 4). The first two principal components were accounted for the total variances of 46.37% and 34.62%, respectively. The contents of heavy metals in the whole crayfish from different geographical environments are quite different, and the distribution of some areas in two-dimensional space is clustered together. Therefore, according to the distribution of different regions, the map of Hubei Province was divided into four groups. The new PCA chart is shown in Figure 4. The whole crayfish from SZ and HH were classified into one group, those from XT, EZ, XG, and QJ into another group, those from ES and SS into yet another group, and those from JX and JY into the final group. The four groups were evenly distributed in the two-dimensional space of PC1 and PC2, indicating that the distribution of heavy metals in each group of the whole crayfish samples had similar characteristics. The geographical spatial distribution demonstrated that the geographical location of each group was very close. XT, EZ, XG, and QJ are all close to the Han River (largest Yangtze tributary) basin in Hubei Province. Therefore, the heavy metal contents of the whole crayfish in these four regions are highly correlated. In particular, there was a strong association between the concentrations of As and Mn in the whole crayfish from JX and JY. A former study of heavy metal pollution of the Wuhan stretch of the Yangtze River [44] specified that the pollution of heavy metals mainly comes from industrial wastewater, electroplating industries, heavy metal processing, and domestic sewage [1]. However, the correlations of Hg and the other eight heavy metals were much lower, perhaps because the main source of Hg pollutant is from the other sources, such as coal combustion.

**Figure 5:**
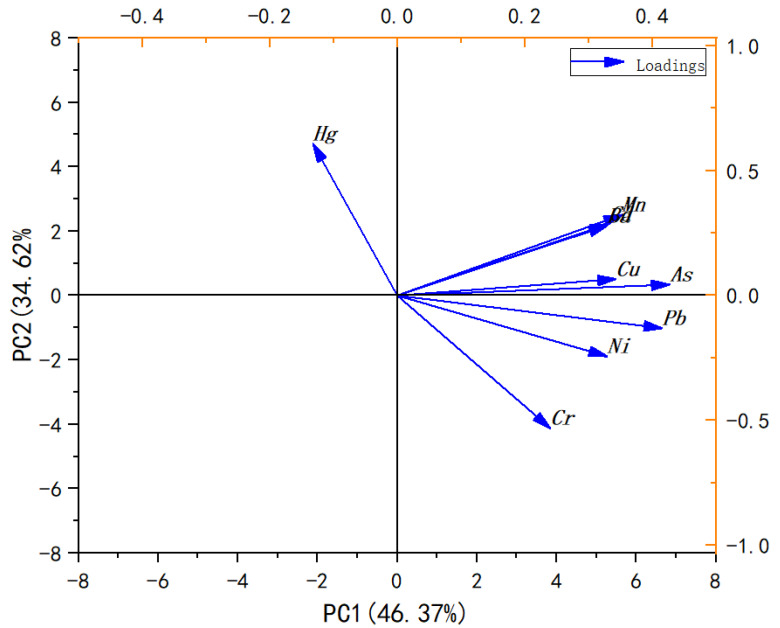
Loading plot of rotated PCA analysis of nine metals in the crayfish samples.

The corresponding results of the rotated component plot regarding the PCA loadings are summarized in Figure 5. In principal component analysis, two principal components with eigenvalues >1 were extracted, comprising 80.99% of the total variance. The two-dimensional diagrams of the first two principal components show that the first component (PC1) accounted for 46.37% of the total variance and presented high loadings for As, Mn, Cd, and Ba. Furthermore, correlations among the metals identified in the multivariate analysis also illustrated the resemblance to the accumulative characteristics of those metals present in aquatic organisms [45]. Nonetheless, PC2, which explained 34.62% of the total variance, showed significant loadings for Hg, Cr, Ni, and Pb, indicating moderate enrichment, which implies that anthropogenic inputs should not be overlooked.

### 3.5. Human Health Risk Assessment

#### 3.5.1. Evaluation of the Estimated Daily Intake

The estimated daily intakes (EDIs) were utilized to estimate both substantial noncarcinogenic risk (THQ) and carcinogenic risk (CR) due to the consummation of the aquatic products [14]. People’s daily exposure to toxic elements through eating foods containing heavy metals was used to avoid any derogative effect on human health over their lifetime [46]. The EDI values of most heavy metals were below the provisional tolerable daily intake (PTDI) based on limits set by the joint FAO/WHO Expert Committee on Food Additives. The PTDI values of As, Cd, Cr, Cu, Hg, Mn, Ni, and Pb are 2.14, 1.0, 3.0, 500, 0.23, 140, 4.26, and 3.57 μg/kg bw/day, respectively [47,48].

Table S4 presents EDI values of heavy metals from the abdominal muscles of crayfish that are consumed by adults and children. The EDI values of most heavy metals were below the provisional tolerable daily intake (PTDI) according to limits set by the joint FAO/WHO Expert Committee on Food Additives [21,47]. For adults, the estimated daily intake for As is (0.3634), Ba (0.6097), Cd (0.0354), Cr (0.7963), Cu (6.2497), Hg (0.0180), Mn (1.8849), Ni (0.2956) and Pb (0.0180). For children, the estimated daily intake for As is (1.5897), Ba (2.6673), Cd (0.1651), Cr (3.7158), Cu (29.1652), Hg (0.0786), Mn (8.7962), Ni (1.3794) and Pb (0.0839). The EDI value is higher for children than adults, which indicates that children are more susceptible to heavy metal exposure [49]. Among the metals selected in this paper, the mean EDI values were basically lower than the PTDI values, which is consistent with a previous conclusion [47]. These results indicate that the heavy metals ingested as a result of crayfish consumption could induce low health risk.

#### 3.5.2. Target Hazard Quotient (THQ)

**Figure 6:**
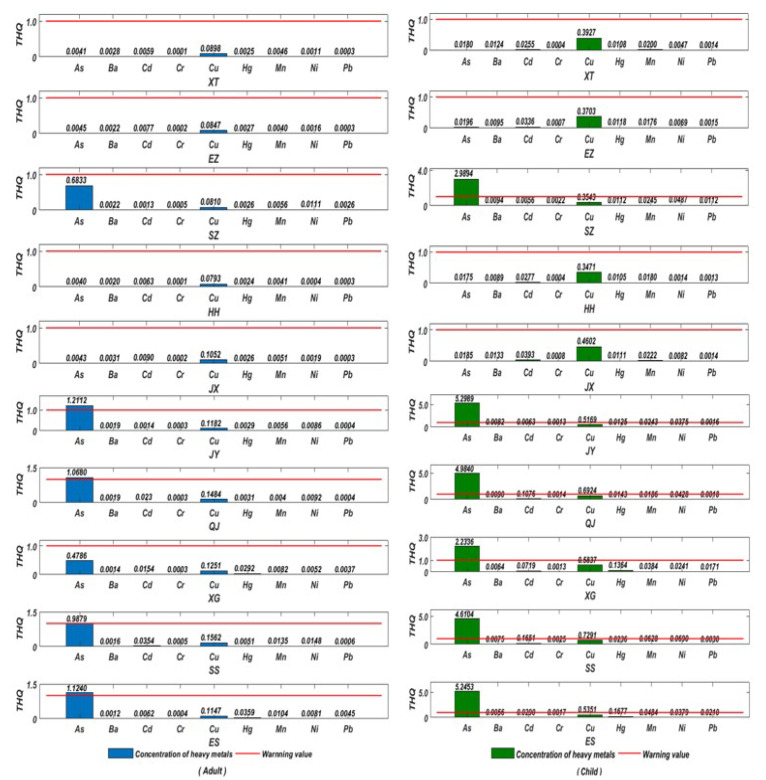
Adult/child hazard risk index of heavy metals in the abdominal muscles of crayfish.

The acceptable threshold limit for THQ is one. When THQ is below the unit limit, it means that the exposure level is lower than the reference dose; thus, exposure to pollutants will have no adverse effect on lifetime consumption [1]. The adult/child hazard risk index of heavy metals in the abdominal muscles of crayfish is presented in Figure 6. The intake of contaminated heavy metals in freshwater crayfish may be a threat to human health [50,51]. The results showed that the average THQ values of As from JY (1.212), QJ (1.068), and ES (1.124) were greater than one for adults, and the THQs of other heavy metals were lower than one. The average THQ values of As from JY (5.297), QJ (4.984), XG (2.234), SS (4.610), and ES (5.245) were greater than one for children, and the THQs of other heavy metals were lower than one, which indicates the adverse health effects on people, especially for children. Consumption of crayfish contaminated with As from the highly contaminated regions showed a higher concentration of heavy metals, signifying adverse health effects on people consuming crayfish from the region [49]. In addition, the presence of Ba, Cd, Cr, Cu, Hg, Mn, Ni, and Pb in the abdominal muscles of crayfish from different areas of the Yangtze River basin may not pose a serious threat to human health [25]. In both adults and children, the THQ values of heavy metals from ingesting crayfish were generally less than one. This suggests that consuming crayfish would not pose significant health risks from taking individual metals [10].

**Figure 7:**
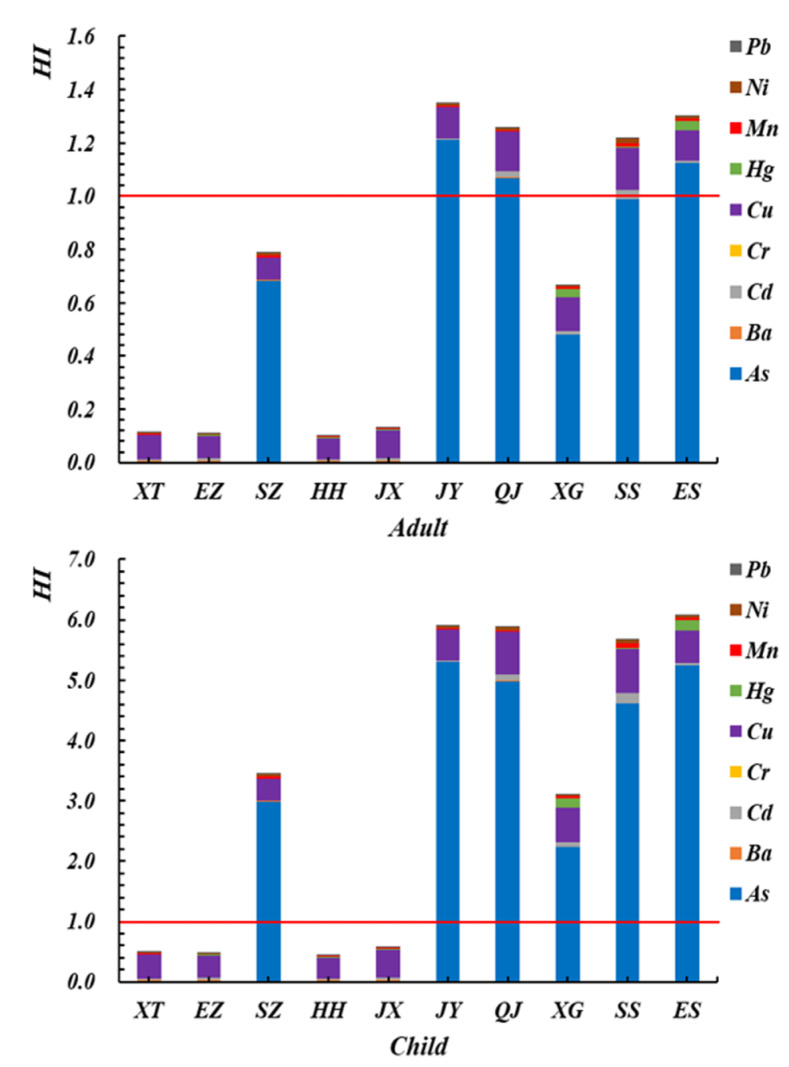
Average HI values of nine heavy metals in crayfish from different areas.

The average HI values of nine heavy metals in crayfish from different areas are included in Figure 7. The average HI values for both adults and children in a single area has consistent with the same ranking trend [30], JY > ES > QJ > SS > SZ > XG > JX > XT > EZ > HH (Figure 7). It is worth noting that the HIs of JY, ES, QJ, and SS for adults were 1.3503, 1.3054, 1.2582, and 1.2150 while those for children were 5.9074, 6.0917, 5.8718, and 5.6730, respectively. These values are greater than one, and the results indicate that crayfish from JY, ES, QJ, and SS in Hubei Province have potential noncarcinogenic effects. In both cases, for adults and children, the target hazard quotient (THQ) was in the order As ˃ Cu ˃ Ni ˃ Mn ˃ Hg ˃ Ba ˃ Cd > Pb > Cr, but the THQ of each metal from the consumption of crayfish was generally less than one. This suggests that consuming such crayfish would not pose significant health risks from the intake of individual metals [10]. In this study, the As concentration contributed considerably to the HI value, accounting for 89.70% of the total noncarcinogenic risk. However, the contributions to the HI value obtained from Cr and Pb were significantly lower than the As level, perhaps only less than 0.03% of the total noncarcinogenic risk. The HI value for children indicates that children in heavy metal-contaminated areas are more vulnerable to adverse effects [30]. This result may be due to particular behavioral patterns in children, such as low resistance and self-harm prevention awareness that is not strong [52].

#### 3.5.3. Estimation of the Cancer Risk (CR)

**Figure 8:**
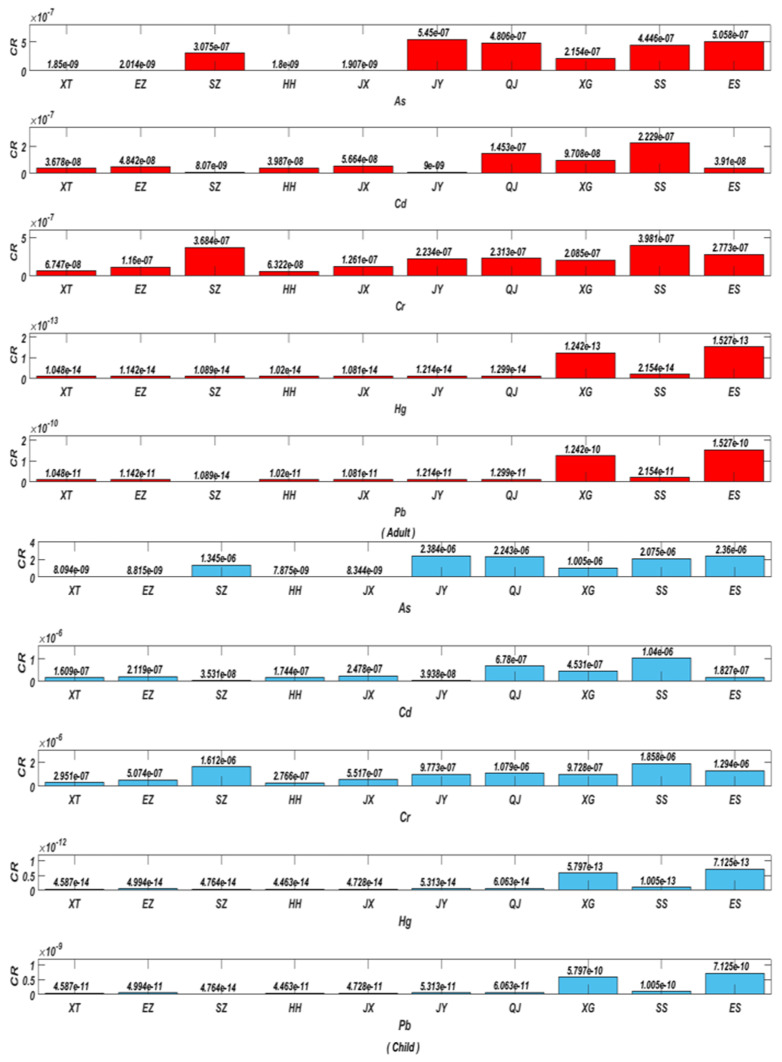
Carcinogenic risk (CR) of heavy metals for different age consumers in the abdominal muscles of crayfish.

The International Agency for Research on Cancer [53] has classified As, Cd, Cr and Pb cause risk of cancer [54] and Hg is classified as ‘possibly carcinogenic’ to humans [54]. The carcinogenic risk (CR) values for As, Cd, Cr, Hg, and Pb were calculated for different ages of consumers in the abdominal muscles of crayfish, and the results are shown in Figure 8. The results suggest that the primary pathway for heavy metal exposure is oral ingestion. The highest mean CR values of crayfish As, Cd, Cr, Hg, and Pb were JY (5.450 × 10^−7^), SS (2.229 × 10^−7^), SS (3.981 × 10^−7^), ES (1.527 × 10^−13^), and ES (1.527 × 10^−10^) for adults and JY (2.386 × 10^−6^), SS (1.040 × 10^−6^), SS (1.858 × 10^−6^), ES (7.125 × 10^−13^) and ES (7.125 × 10^−10^) for children, respectively. In the case of mean exposure levels, the CR values of all heavy metals were between 1 × 10^−6^ and 1 × 10^−4^ and below 1 × 10^−4^, meaning that the cancer risk caused by all heavy metals was also acceptable [9]. It is noticeable that the CR of As and Cd is relatively higher than that of other metals, showing its greater potential to pose carcinogenic risk to people living in JY and SS. In addition, children face a greater risk than adults according to the CR values for other metals, which is similar to the noncarcinogenic risk results of a previous study [30]. Likewise, a higher level of risk of Cd and As was found in the Yangtze River [24]. These results suggest that it is necessary to monitor the toxic metal concentrations in the aquatic organisms for regulatory requirements.

## 4. Conclusions

The concentration of heavy metals from different cities located in the downstream upper Yangtze River was found to be higher than that upstream. Among these, crayfish from the JX and JY areas had the highest mean concentrations of heavy metals. The results of the correlation analysis and PCA illustrate that the major sources of heavy metals in crayfish may originate from metal processing, electroplating industries, industrial wastewater, and domestic sewage. The THQ values of heavy metals show that As in the abdominal muscles of crayfish in ES adults and JY children are (THQ = 1.124 and THQ = 5.297) respectively, and children are more vulnerable to heavy metals than adults and may experience a certain degree of adverse health effects. The CR values for As, Cd, Cr, Hg, and Pb suggested that there was no significant potential carcinogenic risk from crayfish consumption in the middle and lower reaches of the Yangtze River Basin of Hubei Province. Therefore, the risk assessment of heavy metals in crayfish cocultured in rice-crayfish mode in Hubei Province is essential. Through the XPS detection of heavy metal As, it is found that As in crayfish culture environment mainly exists in the form of As^3+^. Therefore, the quality and quantity of crayfish consumption should be moderated to prevent the bioaccumulation of As.

## Data Availability

The datasets generated to obtain the results presented in this article are available from the corresponding authors on reasonable request (hydu@mail.hzau.edu.cn).

## Supplementary Materials

The following are available online at https://www.mdpi.com/article/10.3390/foods11030261/s1, Table S1: Partition information of sampling sites, Table S2: Concentrations of heavy metals (mg kg^−1^ dry weight) in crayfish collected from study sites in Hubei Province, Table S3: Concentrations of heavy metals (mg kg^−1^ dry weight) in abdominal muscles of crayfish collected from study sites in Hubei Province, Table S4: Estimated daily intake (EDI) for an adult/child of heavy metals in abdominal muscles of crayfish (ug kg^−1^ day^−1^).
