# Supplementary material for: Investigation of Bioaccumulation and Human Health Risk Assessment of Heavy Metals in Crayfish (Procambarus clarkii) Farming with a Rice-Crayfish-Based Coculture Breeding Modes
Source: Foods. 2022 Jan 19;11(3):261. doi: 10.3390/foods11030261 (PMC8834495; doi:10.3390/foods11030261)
Supplement: Supplementary file 1 [file foods-11-00261-s001.zip › foods-1506802-supplementary.pdf]

Table S1. Partition information of sampling sites

Serial number	area	Longitude (E) / (°)	Latitude (E) / (°)
1	XT	113.453974	30.364953
2	EZ	114.890593	30.396536
3	SZ	113.373770	31.717497
4	HH	113.475980	29.827000
5	JX	114.313010	30.346530
6	JY	113.939270	29.970540
7	QJ	112.896866	30.421215
8	XG	113.926655	30.926423
9	SS	112.238130	30.326857
10	ES	109.479420	30.295020

Table S2. Concentrations of heavy metals (mg kg^{-1}dry weight) in crayfish collected from study sites in Hubei Province.

Area		Element								
		As	Ba	Cd	Cr	Cu	Hg	Mn	Ni	Pb
XT	Mean	1.37	174.74	0.05	1.41	53.51	0.05	247.11	0.69	0.05
	SD	0.16	36.05	0.00	0.57	14.53	0.00	97.12	0.32	0.00
	CV (%)	11.92	20.63	0.63	40.64	27.15	0.58	39.30	47.04	0.58
EZ	Mean	1.25	236.18	0.06	1.19	57.78	0.05	525.31	0.67	0.05
	SD	0.55	137.96	0.02	0.15	16.40	0.00	405.06	0.32	0.00
	CV (%)	44.13	58.41	35.28	12.76	28.38	0.34	77.11	48.44	0.34
SZ	Mean	3.37	205.30	0.30	1.86	71.39	0.05	537.21	1.02	0.29
	SD	0.79	84.94	0.23	0.36	20.68	0.00	285.24	0.64	0.34
	CV (%)	23.35	41.37	78.55	19.49	28.97	0.70	53.10	63.13	118.24
HH	Mean	3.39	244.70	0.31	1.66	73.17	0.05	504.85	1.01	0.17
	SD	2.32	81.96	0.12	0.28	19.27	0.00	158.12	0.21	0.28
	CV (%)	68.39	33.49	39.99	17.17	26.34	0.28	31.32	20.35	166.31
JX	Mean	4.05	237.14	0.25	7.48	70.71	0.01	559.19	1.42	0.65
	SD	1.33	37.59	0.12	6.66	16.16	0.00	356.24	0.46	0.23
	CV (%)	32.75	15.85	46.93	89.11	22.85	0.00	63.71	32.25	35.85
JY	Mean	3.81	233.92	0.24	2.88	85.62	0.01	538.26	1.60	0.56
	SD	1.66	82.53	0.10	0.83	18.30	0.00	277.37	0.78	0.15
	CV (%)	43.55	35.28	41.47	28.76	21.37	0.00	51.53	48.72	27.15
QJ	Mean	1.77	159.94	0.24	2.44	52.84	0.05	250.53	1.19	0.05
	SD	0.71	55.70	0.09	0.52	11.35	0.00	105.87	0.23	0.00
	CV (%)	40.29	34.83	35.89	21.25	21.47	0.31	42.26	19.26	0.31
XG	Mean	0.67	100.06	0.13	0.49	38.37	0.05	172.57	0.41	0.05
	SD	0.45	21.91	0.03	0.14	3.17	0.00	51.21	0.14	0.00
	CV (%)	67.34	21.90	21.50	28.77	0.83	0.32	29.67	33.60	0.30
SS	Mean	1.82	120.22	0.19	0.86	54.56	0.01	169.22	0.27	0.01
	SD	0.77	49.30	0.07	0.29	11.30	0.00	65.28	0.15	0.00
	CV (%)	42.56	41.01	35.24	33.90	20.71	1.50	38.58	55.71	2.83
ES	Mean	2.04	112.87	0.07	0.72	68.50	0.00	114.07	0.48	0.00
	SD	0.36	14.68	0.02	0.17	10.71	0.00	20.18	0.07	0.00
	CV (%)	17.54	13.00	23.07	24.02	15.64	2.90	17.69	15.44	2.90

Note: CV = coefficient of variation.

SD = standard deviation.

Table S3. Concentrations of heavy metals (mg kg^{-1}dry weight) in abdominal muscles of crayfish collected from study sites in Hubei Province.

Area		Element								
		As	Ba	Cd	Cr	Cu	Hg	Mn	Ni	Pb
XT	Mean	0.00	1.98	0.02	0.47	12.57	0.00	2.24	0.07	0.00
	SD	0.00	1.24	0.01	0.18	2.80	0.00	1.07	0.08	0.00
	CV (%)	13.54	62.63	24.24	38.25	22.25	13.54	47.83	110.26	13.54
EZ	Mean	0.00	1.51	0.03	0.81	11.85	0.00	1.97	0.11	0.00
	SD	0.00	0.51	0.01	0.08	2.10	0.00	0.43	0.10	0.00
	CV (%)	11.26	33.41	30.81	10.02	17.69	11.26	21.82	88.32	11.26
SZ	Mean	0.72	1.50	0.00	2.58	11.34	0.00	2.74	0.78	0.00
	SD	0.17	0.25	0.00	1.02	4.56	0.00	0.71	0.17	0.00
	CV (%)	24.00	16.52	9.92	39.70	40.24	9.92	25.75	21.82	9.92
HH	Mean	0.00	1.42	0.02	0.44	11.11	0.00	2.01	0.02	0.00
	SD	0.00	0.69	0.00	0.03	1.09	0.00	0.71	0.04	0.00
	CV (%)	7.06	48.31	14.05	5.95	9.85	7.06	35.34	196.75	7.06
JX	Mean	0.00	2.13	0.03	0.88	14.73	0.00	2.49	0.13	0.00
	SD	0.00	0.97	0.01	0.42	5.67	0.00	1.33	0.16	0.00
	CV (%)	12.21	45.58	46.38	48.04	38.50	12.21	53.48	124.05	12.21
JY	Mean	1.27	1.31	0.01	1.56	16.54	0.01	2.72	0.60	0.01
	SD	0.36	0.34	0.00	0.22	3.61	0.00	0.51	0.09	0.00
	CV (%)	28.20	25.82	13.74	14.18	21.83	13.74	18.67	14.67	13.74
QJ	Mean	1.12	1.35	0.08	1.62	20.77	0.01	1.95	0.64	0.01
	SD	0.35	0.51	0.04	0.37	8.75	0.00	1.34	0.19	0.00
	CV (%)	31.23	37.73	54.97	22.99	42.10	29.41	68.74	29.79	29.41
XG	Mean	0.50	0.96	0.05	1.46	17.51	0.05	4.03	0.36	0.05
	SD	0.24	0.21	0.01	0.42	2.24	0.01	2.07	0.12	0.01
	CV (%)	48.45	21.48	21.31	28.54	12.78	22.19	51.49	32.82	22.19
SS	Mean	1.04	1.12	0.12	2.79	21.87	0.01	6.60	1.03	0.01
	SD	0.31	0.32	0.05	0.65	2.73	0.00	2.28	0.21	0.00
	CV (%)	30.07	28.40	37.59	23.48	12.49	29.32	34.62	20.58	29.32
ES	Mean	1.18	0.83	0.02	1.94	16.05	0.06	5.09	0.57	0.06
	SD	0.18	0.22	0.01	0.43	2.39	0.01	2.54	0.14	0.01
	CV (%)	15.66	26.52	48.73	22.10	14.88	22.10	49.86	24.84	22.10

Note: CV = coefficient of variation.

SD = standard deviation.

Table S4. Estimated daily intake (EDI) for an adult / child of heavy metals in abdominal muscles of crayfish (ug kg^{-1} day^{-1}).

source	Age	As	Ba	Cd	Cr	Cu	Hg	Mn	Ni	Pb
XT	Adult	0.0012	0.5658	0.0059	0.1349	3.5909	0.0012	0.6392	0.0214	0.0012
	Child	0.0054	2.4754	0.0255	0.5903	15.7102	0.0054	2.7965	0.0934	0.0054
EZ	Adult	0.0014	0.4325	0.0077	0.2320	3.3853	0.0014	0.5634	0.0318	0.0014
	Child	0.0059	1.8920	0.0336	1.0149	14.8105	0.0059	2.4647	0.1390	0.0059
SZ	Adult	0.2050	0.4285	0.0013	0.7368	3.2398	0.0013	0.7842	0.2225	0.0013
	Child	0.8968	1.8745	0.0056	3.2235	14.1740	0.0056	3.4307	0.9737	0.0056
HH	Adult	0.0012	0.4060	0.0063	0.1264	3.1730	0.0012	0.5751	0.0062	0.0012
	Child	0.0053	1.7761	0.0277	0.5531	13.8820	0.0053	2.5159	0.0271	0.0053
JX	Adult	0.0013	0.6097	0.0090	0.2522	4.2077	0.0013	0.7102	0.0374	0.0013
	Child	0.0056	2.6673	0.0393	1.1035	18.4089	0.0056	3.1072	0.1637	0.0056
JY	Adult	0.3634	0.3743	0.0014	0.4467	4.7260	0.0014	0.7761	0.1714	0.0014
	Child	1.5897	1.6378	0.0063	1.9544	20.6763	0.0063	3.3955	0.7499	0.0063
QJ	Adult	0.3204	0.3858	0.0230	0.4625	5.9345	0.0015	0.5564	0.1832	0.0015
	Child	1.4952	1.8004	0.1076	2.1581	27.6943	0.0071	2.5964	0.8551	0.0071
XG	Adult	0.1436	0.2736	0.0154	0.4169	5.0033	0.0146	1.1508	0.1034	0.0146
	Child	0.6701	1.2766	0.0719	1.9455	23.3486	0.0682	5.3703	0.4823	0.0682
SS	Adult	0.2964	0.3199	0.0354	0.7963	6.2497	0.0026	1.8849	0.2956	0.0026
	Child	1.3831	1.4927	0.1651	3.7158	29.1652	0.0118	8.7962	1.3794	0.0118
ES	Adult	0.3372	0.2379	0.0062	0.5546	4.5866	0.0180	1.4533	0.1624	0.0180
	Child	1.5736	1.1102	0.0290	2.5880	21.4042	0.0839	6.7821	0.7578	0.0839
PTDI		2.14	-	1	3	500	0.23	140	4.26	3.57

Note: PTDI: Provisional tolerable daily intake.
